# Characterization of glutamine synthetase involved in the fecundity of *Rhopalosiphum padi*

**DOI:** 10.1038/s41598-025-05567-z

**Published:** 2025-07-01

**Authors:** Xing-Ye Li, Jie-Qiong Wang, Kang-Wu Zheng, Yu-Ting Li

**Affiliations:** 1 College of Plant Protection, Shenyang Agricultural University, Shenyang, 110866 Liaoning China; 2 Key Laboratory of Economical and Applied Entomology of Liaoning Province, Shenyang, 110866 Liaoning China

**Keywords:** Glutamine synthetase, *Rhopalosiphum padi*, Fecundity, Vitellogenin, *Buchnera*, Animal physiology, Entomology

## Abstract

Glutamine synthetase (GS) is a pivotal enzyme crucial for the synthesis of glutamine (Gln), an important precursor in amino acid biosynthesis, essential for the growth, development, and reproduction of insects through its involvement in nitrogen metabolism. Despite its recognized significance in insect biology, the specific functions of GS in aphids have not been fully elucidated. Here, we cloned and characterized two GS genes, *RpGS1* and *RpGS2*, from *Rhopalosiphum padi* and analyzed their expression profiles and explored the contribution of *RpGS* to aphid fecundity. The two isoforms, which are predicted to localize in the mitochondria and cytoplasm respectively, were successfully cloned and heterologously expressed in *Escherichia coli*. Despite exhibiting 92% amino acid similarity, the isoforms displayed distinct enzymatic kinetic properties and demonstrated variations in mRNA expression levels across developmental stages and tissues. Notably, *RpGS1* was highly expressed in the head, whereas *RpGS2* was highly expressed in the intestine. Both *RpGS* genes were significantly expressed in alate adult aphids. Treatment with the specific inhibitor L-methionine S-sulfoximine (MSX) not only suppressed enzyme activity but also downregulated gene expression. Furthermore, inhibition of RpGS led to a marked decrease in the abundance of the obligate symbiont *Buchnera* and reduced the fecundity of *R. padi*. The transcript levels of *RpVg* and *RpGT* were also downregulated. These findings underscore the significant role of *RpGS* in regulating fecundity, suggesting its potential as a target for insecticide development in pest management strategies.

**Supplementary Information:**

The online version contains supplementary material available at 10.1038/s41598-025-05567-z.

## Introduction

Glutamine (Gln), the most abundant nonessential amino acid, plays a crucial role in regulating the proliferation of various cell types^1^. In addition, it serves as a substrate for essential amino acids, nucleotides, proteins, and numerous biosynthetic reactions^2,3^. Glutamine synthetase (GS: EC 6.3.1.2) is found in animals, higher plants, and microorganisms, and utilizes ammonia to convert glutamate (Glu) into Gln by hydrolyzing ATP^4,5^. GS plays an important role in the growth and development of organisms by participating in nitrogen metabolism, and is also an essential detoxifying enzyme in stress and immune responses^6–8^. GS generally consists of three different forms, namely GSI, GSII, and GSIII, of which only GSII has been found in eukaryotes^4,9^.

To date, two different isoforms of GS (mitochondrial GS and cytoplasmic GS) with different structures, kinetic behaviors, and functions have been reported in insects^10^. Both GS isoforms play multiple roles in development, reproduction, and stress responses in insects^7^. For example, GS activity is essential in the early stages of *Drosophila* embryonic development and is involved in the heat shock response^3,11^. miR-4868b is involved in regulating *Nilaparvata lugens* fecundity by targeting *NlGS*, and the expression of *NlGS* is also correlated with vitellogenin (*Vg*) expression^12,13^. In *Bactrocera dorsalis*, mitochondrial *BdGSm* is involved in female fecundity, whereas cytoplasmic *BdGSc* plays a predominant role in larval development and female fecundity^14,15^. GS can also help blood or sap-sucking insects, such as *Aedes aegypti* and *Acyrthosiphon pisum*, to mitigate ammonia toxicity in tissues^16–18^. Sap-feeding insects have evolved symbioses with symbionts to cope with a deficient diet lacking essential amino acids and vitamins^19,20^. GS is upregulated in *A. pisum* bacteriocytes and may participate in the essential amino acid metabolism of the aphid-*Buchnera* partnership^18^. However, the characterization and physiological function of GS in aphids remain ambiguous.

The bird-cherry oat aphid, *Rhopalosiphum padi* (L.), is a globally distributed agricultural pest that causes severe economic losses to wheat crops through direct sap-sucking and transmission of barley yellow dwarf virus (BYDV)^21,22^. With global warming and the frequent occurrence of extreme heat, *R. padi* has become the dominant species among wheat aphids in China^23,24^. However, the overuse of insecticides has led to the development of *R. padi* resistance to some insecticides^25,26^. Therefore, it is necessary to seek new targets for the control of *R. padi*. In this study, we identified and characterized the GS genes from *R. padi* (*RpGS1* and *RpGS2*). Moreover, inhibition of RpGS significantly decreased the abundance of the symbiont *Buchnera* and impacted the fecundity of *R. padi*. Concomitantly, transcript levels of *RpVg* and *RpGT* were also suppressed. The results of the present study can contribute to a deeper understanding of the GS functions and serve as a theoretical foundation for subsequent screening of potential new insecticide targets.

## Results

### Sequence and phylogenetic analysis of *RpGSs*

Two GS isoforms, *RpGS1* (GenBank accession no. OQ434216) and *RpGS2* (GenBank accession no. OQ434217), which are located on two different chromosomes or scaffolds, were isolated and identified in the *R. padi* genome (Fig. S1A). There were eight and seven exons in *RpGS1* and *RpGS2*, respectively (Fig. S1B). Both *RpGSs* contain a glutamine synthetase superfamily domain (PLN02284) (Fig. S1C). The open reading frame (ORF) of *RpGS1* is 1218 bp and encodes 405 amino acids (~ 45 kDa). The ORF of *RpGS2* contains 1128 bp, which encodes 375 amino acids (~ 42 kDa). The isoelectric points of *RpGS1* and *RpGS2* were 6.24 and 6.15, respectively. Both proteins are hydrophilic and have no signal peptide or transmembrane domain (Fig. S2A-C). The prediction of subcellular localization showed that *RpGS1* and *RpGS2* are located in the mitochondria and cytosol, respectively (Fig. S2D).

**Fig. 1:**
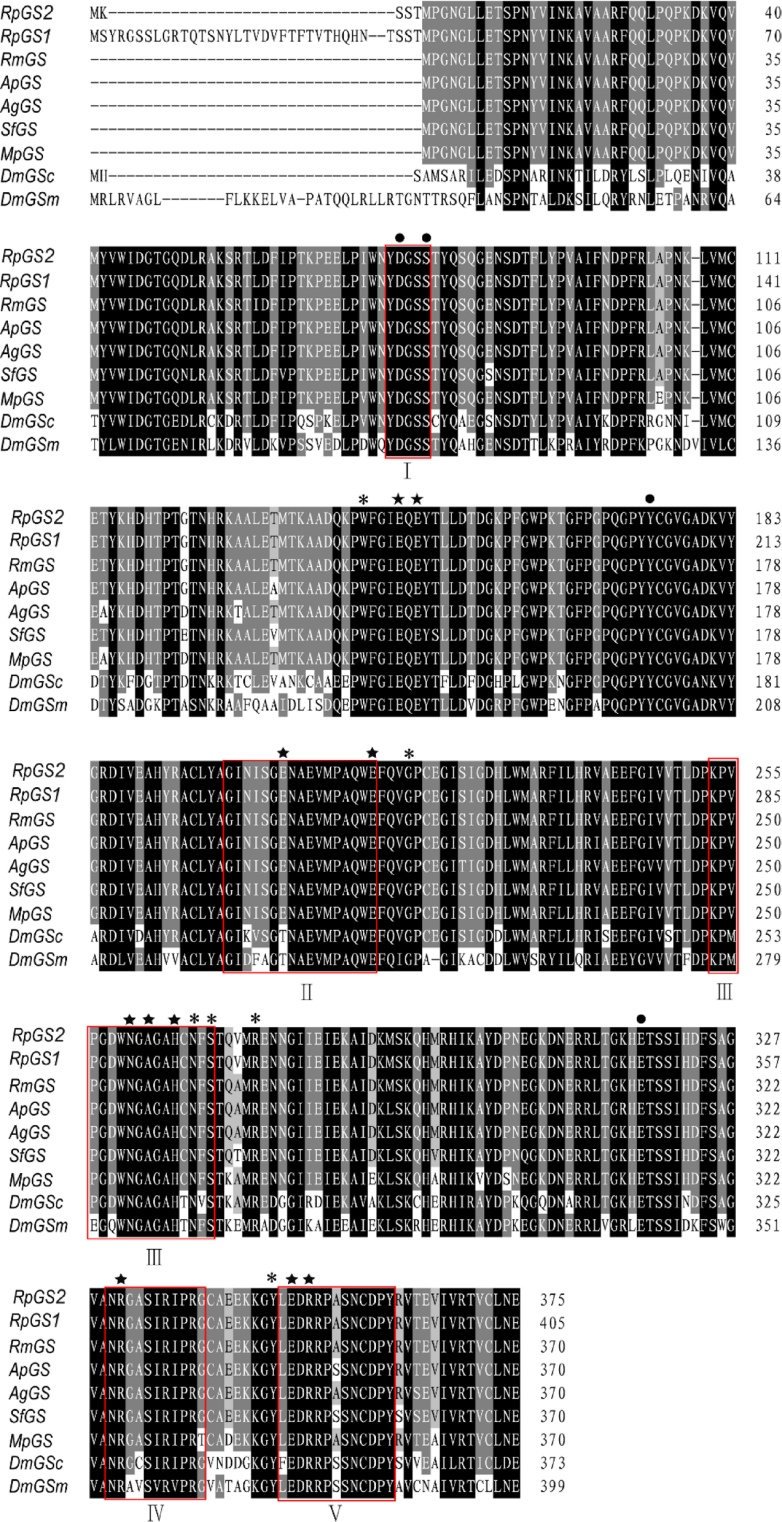
Sequence alignment of the amino acid sequences of GSs from insects. Abbreviations: Rp: *Rhopalosiphum padi*, Rm: *Rhopalosiphum maidis*, Ap: *Acyrthosiphon pisum*, Ag: *Aphis gossypii*, Sf: *Sipha flava*, Mp: *Myzus persicae*, Dm: *Drosophila melanogaster*. The five conserved subdomains are boxed and labeled with Roman numerals(I-V). The NH4^+^-binding residues, ATP-binding residues, and glutamate-binding residues of GS are marked with dots (●), asterisks (*), and pentagrams (★), respectively.

**Fig. 2:**
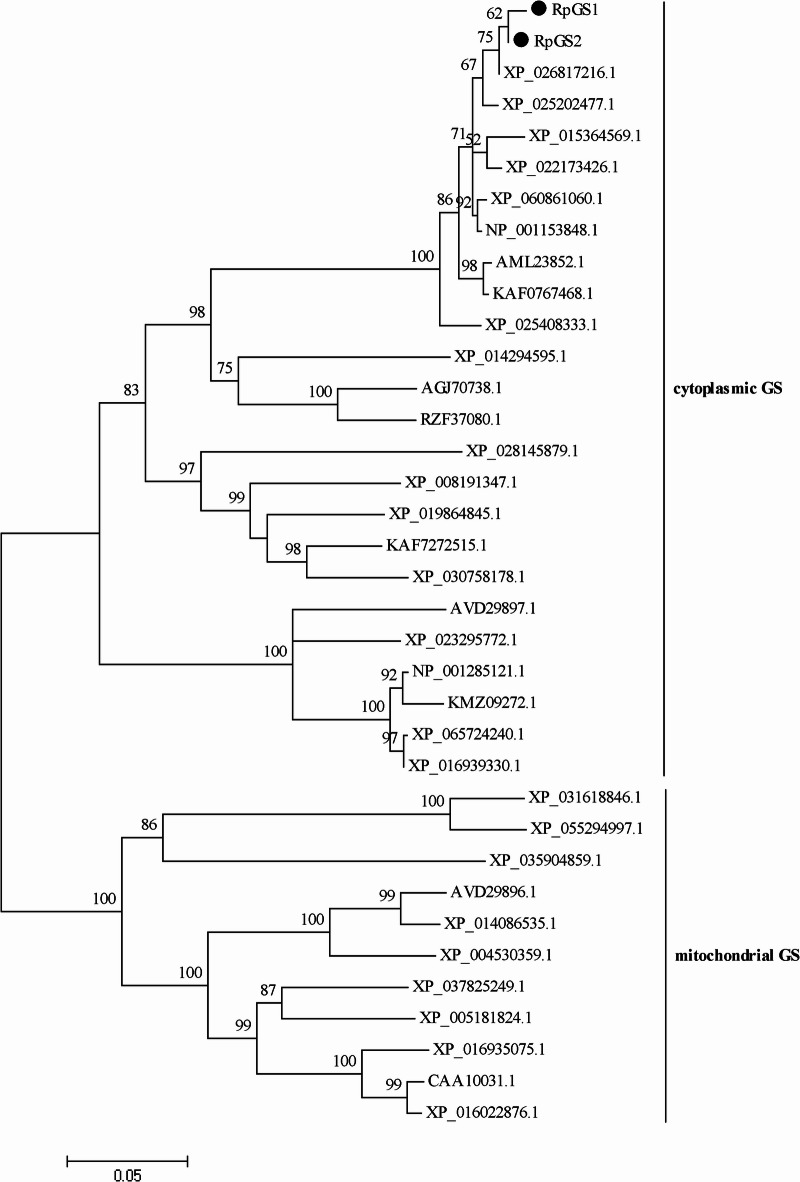
Phylogenetic analysis of *GS* genes from insects. The *GS* genes from *Rhopalosiphum padi* are marked with dots (●). The information for GSs used for phylogenetic analysis is shown in Supplementary Information, Table S2.

BLAST results revealed that the two GS isoforms from *R. padi* were highly conserved with GS from other aphids, and presented 92% amino acid identity with each other. Specifically, RpGS2 shares 98% amino acid identity with the GS of *R. maidis* (XP_026817216.1), whereas RpGS1 shares 96% amino acid identity with the GS of *Diuraphis noxia* (XP_015364569.1)^27^. Furthermore, multiple alignment revealed that both GS proteins contained five conserved regions (Fig. 1). A phylogenetic tree was constructed based on GS sequences from other insects (Fig. 2). The phylogenetic tree of GS is divided into two branches: cytoplasmic GS and mitochondrial GS. The RpGSs were most closely related to the GSs of other aphids.

#### Protein expression, purification and enzyme activity assay

**Fig. 3:**
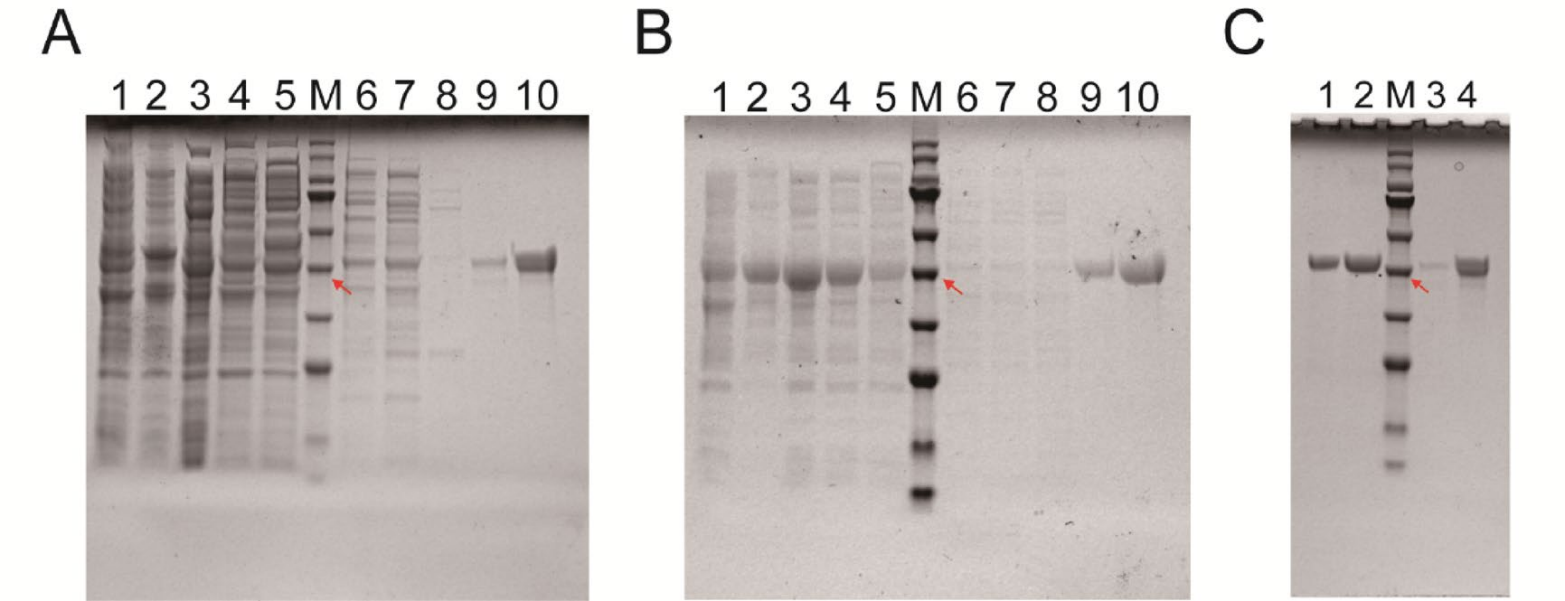
Protein of RpGS expression, and purification. (**A**) SDS-PAGE analysis of recombinant pET-28a-*RpGS1* protein. (**B**) SDS-PAGE analysis of recombinant pET-28a-*RpGS2* protein. A-B, Lane M, protein marker, the red arrow represents 45 kDa. Lane 1, uninduced control; Lane 2, induced bacterial solution; Lane 3, total soluble protein; Lane 4, flow-through fraction; Lane 5, wash-down fraction; Lane 6–10, 50, 100, 150, 200, 250 mM/L imidazole eluate. (**C**) SDS-PAGE analysis of gel-purified recombinant RpGS protein. Lane 1 and 2, purified recombinant pET-28a-*RpGS2* protein from 200 and 250 mM/L imidazole eluate; Lane 3 and 4, purified recombinant pET-28a-*RpGS1* protein from 200 and 250 mM/L imidazole eluate.

Recombinant RpGS proteins were expressed in *E. coli*, and the results showed that both recombinant RpGSs were expressed as soluble proteins (Fig. 3). The SDS-PAGE results revealed that the molecular weight of the recombinant RpGS was close to 45 kDa, which was consistent with the predicted RpGS size (Fig. 3). There are different kinetic parameters under changes in the substrate Glu: the *V*_*max*_ and *K*_*m*_ of RpGS1 are 0.9114 U/mg prot^−1^ and 0.5795 mM, respectively. The *V*_*max*_ and *K*_*m*_ of RpGS2 are 0.7175 U/mg prot^−1^ and 0.5301 mM, respectively (Table 1).

**Table 1:** Kinetic properties of RpGS.

	RpGS1	RpGS2
V_max_* (U mg^−1^ prot^−1^ 95%CI)	0.9114 (0.8550–0.9721)	0.7175 (0.6780–0.7597)
K_m_ (mM 95%CI)	0.5795 (0.3726–0.8654)	0.5301 (0.3521–0.7691)

Note: *1U indicates a 0.005 change in the absorbance value at 540 nm per mg of protein per min in the reaction system.

#### Relative expression profiles of *GSs* in *R. padi*

**Fig. 4:**
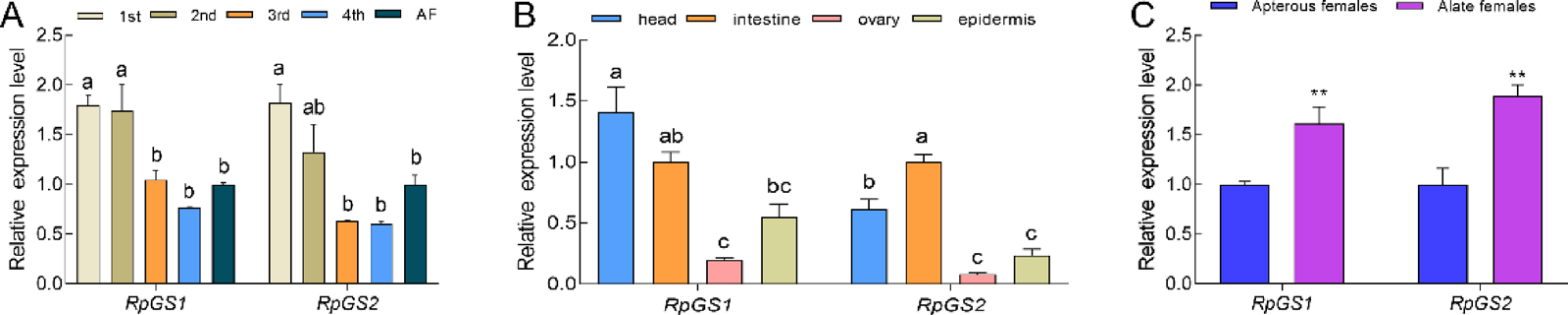
Relative expression profiles of GSs in ***Rhopalosiphum padi***. The relative expression of *RpGSs* at different developmental stages (A), in different tissues (B), and in the wing dimorphis (C) of *Rhopalosiphum padi*. Data represent the means ± S.E. Different letters or asterisks on the bars indicate significant differences by Tukey-HSD multiple comparison (*P* < 0.05) and *t* test pairwise comparison (**P < 0.01), respectively.

The expression patterns of the two *RpGSs* in different developmental stages, tissues and wing dimorphisms of *R. padi* were determined by RT-qPCR (Fig. 4). The results revealed that both genes were expressed throughout all developmental stages, with particularly high expression levels in the 1 st instar nymphs (Fig. 4A). There were no significant differences in the relative expression levels of *RpGSs* among the 3rd instars, 4th instars, and adults (Fig. 4A). Both *RpGSs* were ubiquitously expressed in all the tested tissues, with the highest expression levels of *RpGS1* observed in the head and *RpGS2* in the intestine (Fig. 4B). However, the relative expression levels of both genes were lower in the ovary than in the other tissues. The mRNA transcript levels of *RpGS1* and *RpGS2* were significantly elevated in alate adults compared with those in apterous adults, indicating a wing morph-related expression pattern (Fig. 4C).

#### Analysis of the role of *RpGSs* in aphid fecundity and the *Buchnera* titer

**Fig. 5:**
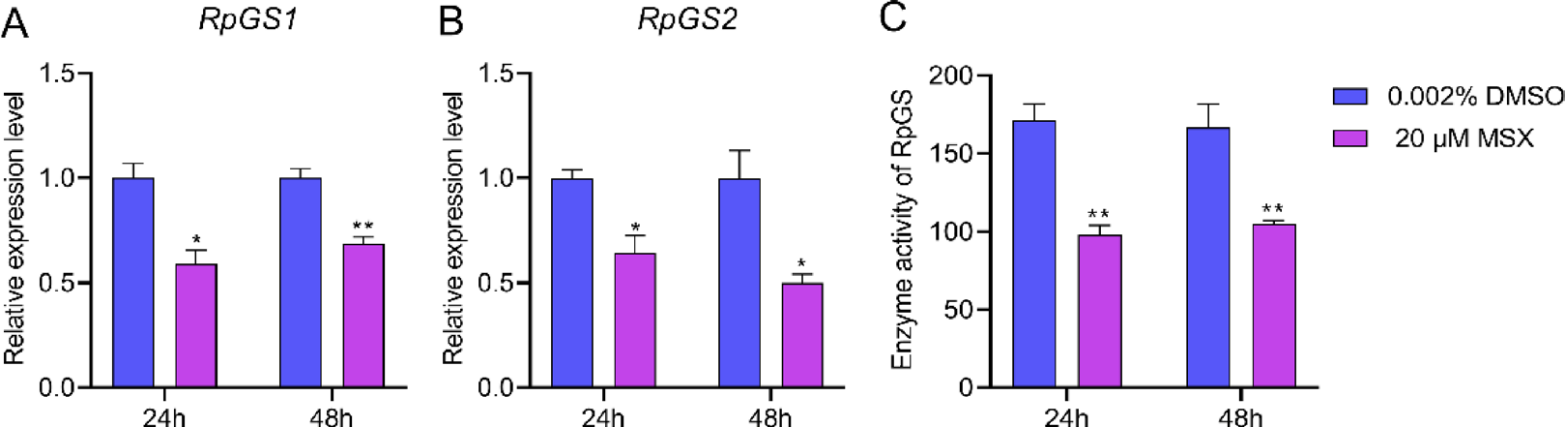
Inhibition efficiency of MSX. Relative expression levels of *RpGS1* (A) and *RpGS2* (B) and the activity of GS enzyme (C) in *Rhopalosiphum padi* injected with 20 µM MSX. The data are represented as the means ± S.E. Asterisks on the bars indicate significant differences between treated and control groups (*P < 0.05, **P < 0.01).

**Fig. 6:**
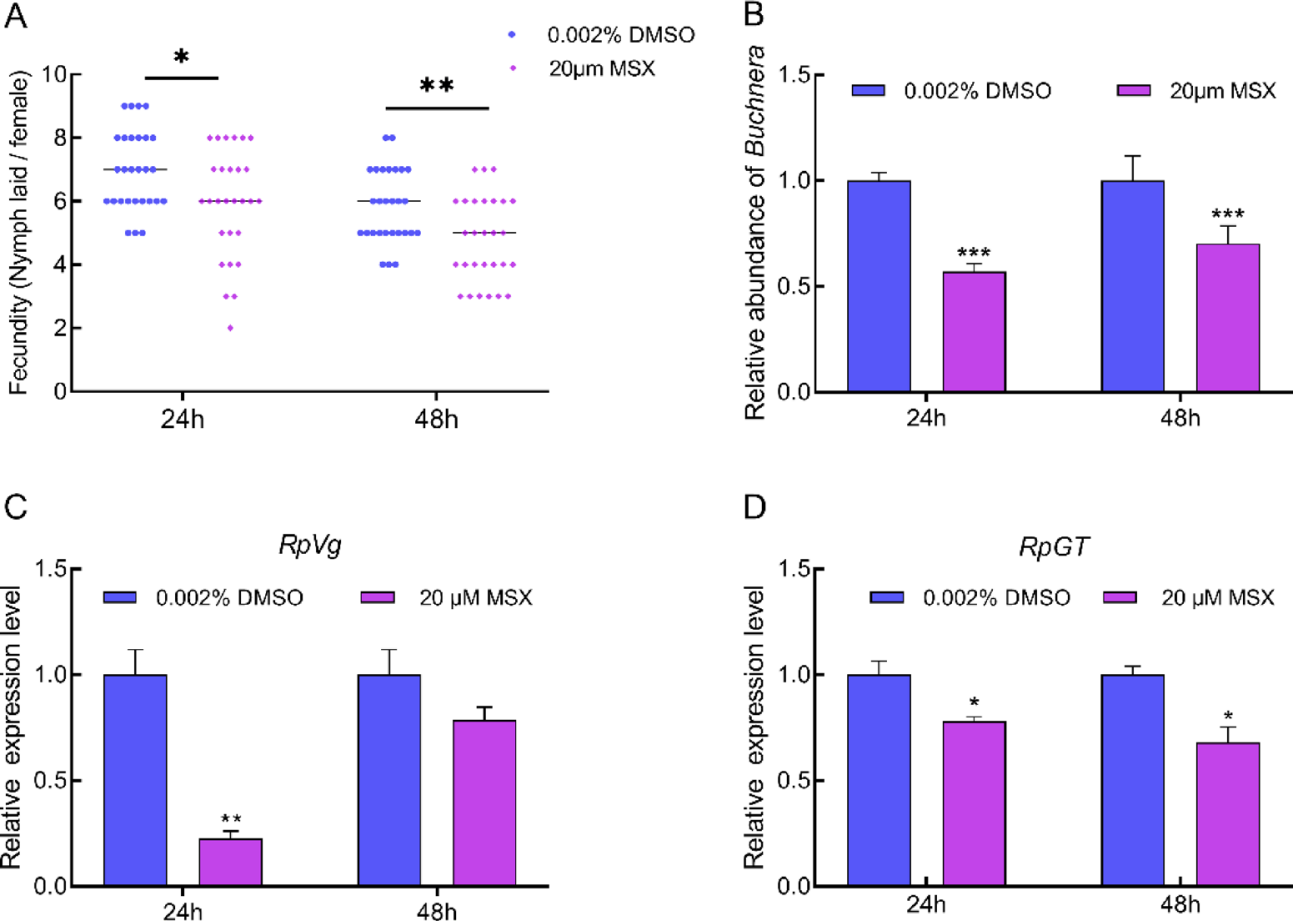
Analysis of *RpGS* in aphid reproduction by the inhibitor MSX. The fecundity (**A**), *Buchenera* titer (**B**), and relative expression levels of *RpVg* (C) and *RpGT* (D) in *Rhopalosiphum padi* injected with 20 µM MSX. The data represent the means ± S.E. Asterisks represent significant differences between treated and control groups (*P < 0.05, **P < 0.01, ***P < 0.001).

To verify the function of RpGSs, a specific inhibitor (MSX) of GS was applied, resulting in significant decreases in the expression levels of *RpGS1* and *RpGS2* of 41% (*P* = 0.013) and 32% (*P* = 0.005) at 24 h, and 36% (*P* = 0.018) and 50% (*P* = 0.022) at 48 h post injection (Fig. 5A-B). The GS enzyme activity also decreased by 43% (*P* = 0.001) and 37% (*P* = 0.006) at 24 and 48 h after MSX injection, respectively (Fig. 5C). Injection of MSX led to an 8.5% (*P* = 0.012) reduction in reproduction at 24 h post-injection and an 8.2% (*P* = 0.003) reduction at 48 h post injection (Fig. 6A). Compared with that of the control, the abundance of *Buchnera* significantly 43% (*P* < 0.05) and 29.7% (*P* < 0.05) lower at 24 h and 48 h post-MSX injection, respectively (Fig. 6B). Compared with the control, a significant decrease of 77.3% (*P* = 0.003) in *RpVg* expression was observed at 24 h post injection, whereas an insignificant decrease of 21.5% was observed at 48 h post injection (Fig. 6B). In addition, the transporter *RpGT*, which is responsible for transporting GS into bacteriocytes, exhibited significant decreases of 22% (*P* = 0.03) and 32% (*P* = 0.02) at 24 h and 48 h post-MSX injection, respectively (Fig. 6D).

## Discussion

GSs are described as the oldest and highly conserved functional genes, involved in several biological processes in organisms, including reproduction, growth and development^4,6^. However, the specific role of GS in *R. padi* has not been previously reported. In this study, two different GS isoforms were successfully isolated and identified from *R. padi* and expressed separately in vitro. The expression patterns of *RpGS1* and *RpGS2* were found to be affected by developmental stage, wing morph, and different tissues. Furthermore, the decreased expression and enzyme activity of *RpGSs* resulted in a significant decline in *R. padi* reproduction, the expression levels of *RpVg* and the abundance of *Buchnera*. Thus, understanding the precise role of *RpGSs* in reproduction is vital for the development of environmentally friendly pest control strategies.

GS serves as a molecular clock for determining phylogenetic relationships among various species. It is recognized as the oldest extant and functional gene in evolutionary history^4^. In this study, the RpGSs were successfully cloned and sequenced, with the GenBank accession numbers OQ434216 and OQ434217. Both RpGSs contain the glutamine synthetase superfamily domain (PLN02284) and five conserved subdomains, confirming their classification within the GSII group. Notably, RpGS2 and RpGS1 exhibit 92% amino acid identity, which is greater than that observed in *B. dorsalis*^14^. Despite this high similarity, predictions regarding subcellular and chromosomal localization suggest that RpGS2 and RpGS1 are distinct isoforms. Further experiments are necessary to clarify the reasons behind the high identity between these two genes in *R. padi*.

The catalytic activity of GS is contingent upon the functionality of the GS protein. In this study, recombinant RpGSs were successfully expressed as soluble proteins in the BL21 (DE3) strain of *E. coli*, which aligns with previous findings in *Leishmania donovani*^28^. The molecular weight of RpGSs was comparable to that of GSs in *D. melanogaster* (42 kDa)^10^ and *Apis cerana cerana* (~ 45 kDa)^7^. The purified fusion protein exhibited significant hydrolase activity against the substrate Glu, with *K*_*m*_ values of 0.5795 mM for RpGS1 and 0.5301 mM for RpGS2 (Table 1). These results suggest that RpGS1 and RpGS2 have distinct kinetic parameters, indicating the potential for different functional properties.

The expression of GS genes is influenced by developmental stage and tissue^14,29^. Our study revealed that both *RpGS* isoforms are expressed throughout all developmental stages, suggesting their involvement in overall development^5,30^. Notably, *RpGS1* exhibited high expression in the head, whereas *RpGS2* showed elevated expression in the intestine. This pattern of differential expression is also observed in other species; for example, two GS isoforms in *D. melanogaster*^30^ and *B. dorsalis*^14^ display distinct expression patterns similar to those in *R. padi*. In *Aedes aegypti*, the midgut efficiently incorporates ammonia into amino acids through specific metabolic pathways^17^. Additionally, high expression of GS in head and neural tissues has been reported in *Apis cerana*^7^*B. dorsalis*^14^*A. aegypti*^31^and *Schistocerca gregaria*^32^. These findings indicate that the two GS isoforms may play different functional roles in insects. Smartt et al.. (1998) noted that mosquitoes experience paralysis and are unable to fly when GS activity is inhibited^29^. Interestingly, both *RpGS2* and *RpGS1* were highly expressed in alate aphids, indicating that GS may play a crucial role in the maintenance of flight^33^.

Previous studies manifested that GS plays a crucial role in insects, particularly in terms of female fecundity^14^. GS regulates female reproduction through various mechanisms, including vitellogenin synthesis, ovarian development, and the TOR pathway^12,13,34^. In our study, we inhibited the activity of GS and the expression of *RpGSs* by injecting adults with the GS-specific inhibitor, MSX. This intervention led to a significant reduction in both fecundity and vitellogenin (*Vg*) expression in *R. padi* when the expression of both RpGS genes and enzyme activity were suppressed. GS can exclusively catalyze the synthesis of Gln, which is the predominant amino acid in the hemolymph of aphids and is essential for the biosynthesis of both nonessential and essential amino acids^35,36^. In *A. pisum*, GS is upregulated in bacteriocytes, whereas Gln is imported into these cells via a glutamine transporter^18,36^. Our findings indicate that the inhibition of RpGSs resulted in a significant decrease in the abundance of *Buchnera* and in the transcript levels of the glutamine transporter gene (*RpGT*). This suggests that the decline in Gln synthesis due to GS inhibition may lead to reduced *Buchnera* abundance, resulting from nutrient deficiency caused by the lack of Gln, a precursor for amino acid synthesis^37,38^. In summary, nutritional symbionts such as *Buchnera* in aphids are regulated by nutrient availability and are critical for the reproduction of aphids^19,39,40^.

Overall, two *RpGSs* were successfully cloned and demonstrated stable expression in *R. padi* in a developmental or tissue-specific manner. The application of the GS-specific inhibitor MSX significantly reduced the enzyme activity and expression level of *RpGSs*. This inhibition of RpGSs led to a marked decrease in *Buchnera* abundance and fecundity. Additionally, the transcript levels of *RpVg* and *RpGT* were downregulated. These findings suggest that *RpGSs* may serve as potential targets for future technologies aimed at controlling *R. padi* and offer new insights into the regulatory mechanisms of fecundity in aphids.

## Materials and methods

### Insects

A culture of *R. padi* provided by Northwest Agriculture and Forestry University and collected from wheat in Shaanxi Province, was reared on wheat (*Triticum aestivum* L.) cv. Changfeng 2112. *R. padi* were maintained in climate-controlled chambers at 24 ± 1 °C with a 16 h light: 8 h dark regime.

Newly born nymphs (1st instar), newly molted nymphs (2nd, 3rd, and 4th instars), and 2-day-old wingless adults were used for the analysis of *RpGS* expression at different developmental stages. Two-day-old apterous and alate adults were selected for analysis of *RpGS* expression in different wing morphs. The head, gut, ovary and cuticle tissues from 2-day-old wingless adults were dissected in phosphate-buffered solution (PBS, pH = 7.2) to analyze the expression of *RpGSs* in different tissues. The aphids and tissues were frozen immediately in liquid nitrogen and stored at − 80 °C until use. Each developmental stage, wing morph and tissue included three replicates.

## RNA isolation and cDNA synthesis

Total RNA was extracted from each treatment using TRIzol reagent (Invitrogen, CA, USA), following the manufacturer’s instructions. The integrity and concentration of the obtained RNA were determined using a NanoDrop2000 spectrophotometer (Thermo Fisher Scientific, MA, USA), respectively. Prior to cDNA synthesis, the RNA samples were treated with RQ1 RNase-Free DNase (Promega, Madison, WI, USA), to eliminate genomic DNA. First-strand cDNA was synthesized using 2 µg of total RNA using the GoScript™ Reverse Transcription System (Promega, WI, USA) according to the manufacturer’s instructions.

### Molecular cloning of *RpGSs*

The amino acid sequence of the GS from *Drosophila melanogaster* (GenBank accession number: CAA10031) was used as a query sequence in the *R. padi* genome (GCA_019425515.1), and two candidate genes were screened. Primers designed using Primer Premier 5.0 (Premier Biosoft International, CA, USA) were used to amplify the ORF of *RpGSs* (Table S1). PCR was performed in a mixture including 2.0 µL cDNA template, 10 µL 2×Taq Master Mix (Vazyme, Nanjing, China), 0.8 µL each primer (10 μm/L), and 6.4 µL nuclease-free water in a total volume of 20 µL. The PCR cycling parameters were as follows: initial denaturation at 92 ℃ for 3 min, 35 cycles at 92 ℃ for 30 s, 58 ℃ for 30 s, and 72 ℃ for 90 s; and a final extension at 72 ℃ for 10 min. The PCR products were purified from 1% agarose gels by the Wizard PCR Preps Kit (Promega, WI, USA). The purified fragment was subsequently cloned and inserted into a pMD19-T vector (Takara, Beijing, China) and transformed into *Escherichia coli* DH5α-competent cells. Positive clones were selected and sequenced (Sangon Biotech Co. Ltd., Shanghai, China).

### Bioinformatics and phylogenetic analysis of *RpGSs*

Homology searches for nucleotide and amino acid sequences were conducted using the BLAST program of the National Center for Biotechnology Information (NCBI) (https://blast.ncbi.nlm.nih.gov/Blast.cgi). Multiple comparisons were performed using MAFFT v7.487^41^. The sequence comparison results were embellished using GeneDoc 2.7. Protein subcellular localization was predicted using WoLF PSORT prediction (http://www.genscript.com/wolf-psort.html). Conserved domains were searched on the SMART website (http://smart.embl-heidelberg.de/). The theoretical isoelectric point and molecular weight were calculated using SWISSPROT (ExPASy server) tool (http://web.ExPASy.org/compute_pi/). SignalP 6.0 (https://services.healthtech.dtu.dk/service.php? SignalP) was used to predict the signal peptides. Transmembrane helices were analyzed on the TMHMM Server v.2.0 (http://www.cbs.dtu.dk/services/TMHMM-2.0/). Hydrophobicity was estimated using ProtScale (http://web.ExPASy.org/protscale/). The phylogenetic tree was constructed with the neighbor-joining (NJ) method using MEGA X and 1000 bootstrap analyses.

### Protein expression/purification and Western blot analysis

The recombinant proteins were expressed and purified as described in Wang et al.. (2019)^42^. The verified fragments were subsequently cloned and inserted into the expression vector pET-28a (+) and transformed into *E. coli* BL21 (DE3) cells for protein expression. The recombinant proteins were identified by 12% SDS-PAGE with standard protein-sized markers (Thermo Scientific, Waltham, MA). The concentration of the recombinant proteins was determined using a BCA protein assay kit (Songon Biotech, Shanghai, China).

Twenty microliters of the purified protein sample were separated by 12% SDS-PAGE and subsequently transferred to a nitrocellulose membrane for 90 min at a constant current of 300 mA. The membrane was then blocked for 2 h at room temperature using 5% skim milk in PBS containing 0.1% Tween-20. Detection of the purified proteins was performed using a rabbit anti-His-tag monoclonal antibody (diluted 1:500) followed by incubation with horseradish peroxidase (HRP)-conjugated goat anti-rabbit IgG antibody (Beyotime Biotechnology, Shanghai, China; diluted 1:3000). Chemiluminescent signals were visualized using an ECL detection kit, and images were captured with the Tanon-5200 Multi Chemiluminescent Imaging System (Shanghai, China).

### Measurement of GS enzyme activity

Enzyme activity was measured using the method described by Zhai et al.. (2015)^34^with some modifications. The crude enzyme was prepared using an extraction solution (50 mM Tris, 2 mM MgSO_4_∙7H_2_O, 2 mM DTT, 400 mM sucrose, and 2 mM EGTA, pH 8.0). For the test reaction mixture, 160 µL of solution B (80 mM hydroxylamine hydrochloride, 100 mM Tris, 80 mM MgSO_4_∙7H_2_0, 20 mM sodium glutamate, 20 mM L-cysteine, and 2 mM EGTA, pH 7.4) was mixed with 40 mM ATP and 70 µL of crude enzyme. The control reaction mixture was the same, but solution B did not contain 80 mM hydroxylamine hydrochloride. All reaction mixtures were incubated at 37℃ for 30 min and then stopped by adding 100 µL of color agent (0.2 M TCA, 0.37 M FeCl_3_∙6H_2_O, 0.6 M concentrated hydrochloric acid) was added, and the mixture was left to stand for 10 min at room temperature. After centrifugation for 10 min, the absorbance of the supernatant was measured at 540 nm against a reagent blank.

A recombinant bacterial sample was obtained from 200 ml of bacterial solution through centrifugation, followed by resuspension in extraction reagent. The recombinant protein was then released by ultrasonication, and the resulting protein was used for subsequent enzymatic activity testing. The inactivated protein was used as a control. The enzymatic kinetics of the recombinant protein were measured by adding reaction solution B containing different concentrations of sodium glutamate (0.5, 1, 3, 5, 7, 9, 11, 13, and 15 mM). The values of K_m_ and V_max_ were determined by plotting Michaelis-Menten curves.

### Effect of specific inhibitors on *RpGS*

L-Methionine S-sulfoximine (MSX) is a GS-specific inhibitor that can irreversibly block the catalytic activity of GS. According to the preliminary test results (Fig. S5), 50 nL MSX (20 µM) was injected into newly emerged wingless aphids using the Eppendorf microinjection system, and the inhibitory effect on the expression of the two *RpGS* genes was tested at 24 h and 48 h post injection. DMSO (0.002%) was used as control. In total, 250 aphids were injected. Ten and five aphids were randomly selected post injection for RT-qPCR and enzyme activity measurement, respectively. RT-qPCR was repeated three times, and enzyme activity was measured four times.

### Effects of MSX on fecundity and *Buchnera *titer

To investigate whether the inhibition of *RpGS* affects fecundity and obligate symbiont *Buchnera* titers, ~ 100 newly emerged apterous adults were microinjected with MSX. The injected aphids were reared individually in a small device with fresh wheat leaves (1.5 × 1.5 cm). The wheat leaves were placed on 1% agar gel and replaced every 24 h. Fecundity were recorded at 24 h and 48 h post injection. DNA was extracted from individual adult for each of six biological replicates at 24 h and 48 h post injection. The *Buchnera* titers were determined by quantitative PCR (qPCR) method and calculated the ratio of the copy number of the *Buchnera* 16 S rRNA gene to aphid *β*-actin gene.

### Quantitative PCR (qPCR) and real-time quantitative PCR (RT-qPCR)

Total DNA was extracted following the nonidet-P40-based protocol of Luan et al. (2018)^43^. Each 20 µL reaction mixture consisted of a 2.0 µL cDNA or DNA template, 10 µL SYBR mix (Bimake, USA), 0.8 µL each primer (10 µmol/L), and 6.4 µL nuclease-free water. A melting curve was determined (ramping from 65 °C to 95 °C at 0.5 °C every 5 s) to confirm the amplification of the specific PCR products. The *R. padi β*-actin gene (GenBank: KJ612090.1) was used as the internal control (Table S1). The qPCR and RT-qPCR were performed using a CFX96 Real-Time PCR Detection System (Bio-Rad). *Buchnera* titers and relative expression levels were calculated using the 2^−ΔCt^ and 2^−ΔΔCt^ methods, respectively^44^. Experiments were repeated in three biological replicates, and each replicate was performed at least three times.

### Statistical analyses

Multiple comparisons of *RpGS* gene expression among tissues and developmental stages were performed using one-way ANOVA with Tukey’s test (*P* < 0.05). The independent samples *t tests* (*P* < 0.05) were used to compare two samples. All data analyses were conducted using the Statistica version 12 software (StatSoft). All results were plotted using GraphPad 8.0 (GraphPad Software, CA, USA).

## Electronic supplementary material

Below is the link to the electronic supplementary material.

Supplementary Material 1

Supplementary Material 2

## Data Availability

The sequences generated during the current study are available in the NCBI repository [https://www.ncbi.nlm.nih.gov/, OQ434216, OQ434217]. All the data generated in this experiment are presented in the manuscript and its supplementary information.
