# Supplementary material for: Characterization of glutamine synthetase involved in the fecundity of Rhopalosiphum padi
Source: Sci Rep. 2025 Jul 1;15:20461. doi: 10.1038/s41598-025-05567-z (PMC12216525; doi:10.1038/s41598-025-05567-z)
Supplement: Supplementary file 1 — Supplementary Material 1 [file 41598_2025_5567_MOESM1_ESM.docx]

**Supplementary information**

Characterization of glutamine synthetase involved in fecundity of *Rhopalosiphum padi*

Xing-Ye Li^1,2^, Jie-Qiong Wang^1,2^, Kang-Wu Zheng^1,2^, Yu-Ting Li^1,2^ *

^1^ College of Plant Protection, Shenyang Agricultural University, Shenyang 110866, Liaoning, China.

^2^ Key Laboratory of Economical and Applied Entomology of Liaoning Province, Shenyang 110866, Liaoning, China.

*Correspondence:

Yu-Ting Li, PhD

College of Plant Protection, Shenyang Agricultural University, Shenyang 110866, Liaoning, China. Tel: +86 024 88487148; email: [yutingli2017@syau.edu.cn](mailto:yutingli2017@syau.edu.cn); ORCID: 0000-0003-3357-7554

**Table S1**. Primers used in this study

| Primer name | Sequence（5‘-3’） | Application | Reference |
| --- | --- | --- | --- |
| *GS1-F* | ATGTCATATCGTGGTTCTTCT | Full-length | In this study |
| *GS2-F* | ATGAAATCTTCAACCATGCC | Full-length | In this study |
| *GS-R* | TTATTCGTTTAGGCATACAGT | Full-length | In this study |
| *Bam HI-1F* | CGCGGATCCATGTCATATCGTGGTTCTTCT | Protein expression | In this study |
| *Bam HI-2F* | CGCGGATCCATGAAATCTTCAACCATGCC | Protein expression | In this study |
| *Hind III-R* | CCCAAGCTTTTATTCGTTTAGGCATACAGT | Protein expression | In this study |
| *qActin-F* | GCCCAATCCAAAAGAGGTAT | RT-qPCR | Balakrishnan *et al*., 2018 |
| *qActin-R* | TCAAAGGTGCTTCCGTTAGT | RT-qPCR | Balakrishnan *et al.*, 2018 |
| *qGS1-*F | GCAACTACCTGACAGTCGACGTT | RT-qPCR | In this study |
| *qGS2-*F | ATGAAATCTTCAACCATGCCGGGA | RT-qPCR | In this study |
| *qGS-*R | TCCGTCGATCCAAACATACATGACT | RT-qPCR | In this study |
| *qVg-*F | AGAGACGATGCTCGCACTGAAA | RT-qPCR | Fan *et al*., 2020 |
| *qVg-*R | TTCTGGAAGACATTGGGGAAGTAT | RT-qPCR | Fan *et al*., 2020 |
| *qBuch-F* | CACACTGGAACTGAGACACG | qPCR | Liu *et al*., 2023 |
| *qBuch-R* | CTTCTTCATACACGCGGCAT | qPCR | Liu *et al*., 2023 |
| *qGT-F* | AGTTCGGTGGCTACAATCCA | RT-qPCR | In this study |
| *qGT-R* | GCATGGCCAATATGCCTGAA | RT-qPCR | In this study |

**Reference**

Balakrishnan, B., Su, S., Wang, K., Tian, R. Z., Chen, M. H. Identification, expression, and regulation of an omega class glutathione S-transferase in *Rhopalosiphum padi* (L.) (Hemiptera: Aphididae) under insecticide stress. *Front. Physiol.* **9**, 427. <https://doi.org/10.3389/fphys.2018.00427> (2018).

Fan, Y. J., Li, X. X., Mohammed, A. A. A. H., Liu, Y., & Gao, X. W. miR-147b-modulated expression of vestigial regulates wing development in the bird cherry-oat aphid *Rhopalosiphum padi*. *BMC Genomics* **21**(1), 71. <https://doi.org/10.1186/s12864-020-6466-7> (2020).

Liu, S. *et al*. 2023. Secondary symbionts affect aphid fitness and the titer of primary symbiont. *Front. Plant Sci*. ***14***, 1096750. <https://doi.org/10.3389/fpls.2023.1096750> (2023).

**Table S2.** Information for GS used for phylogenetic analysis.

| **GS** | **Insect species** | **GenBank No.** |
| --- | --- | --- |
| *Cytoplasmic GS* | *Rhopalosiphum maidis* | XP_026817216.1 |
|  | *Melanaphis sacchari* | XP_025202477.1 |
|  | *Diuraphis noxia* | XP_015364569.1 |
|  | *Myzus persicae* | XP_022173426.1 |
|  | *Metopolophium dirhodum* | XP_060861060.1 |
|  | *Acyrthosiphon pisum* | NP_001153848.1 |
|  | *Aphis gossypii* | AML23852.1 |
|  | *A. craccivora* | KAF0767468.1 |
|  | *Sipha flava* | XP_025408333.1 |
|  | *Halyomorpha halys* | XP_014294595.1 |
|  | *Nilaparvata lugens* | AGJ70738.1 |
|  | *Laodelphax striatellus* | RZF37080.1 |
|  | *Diabrotica virgifera virgifera* | XP_028145879.1 |
|  | *Tribolium castaneum* | XP_008191347.1 |
|  | *Aethina tumida* | XP_019864845.1 |
|  | *Rhynchophorus ferrugineus* | KAF7272515.1 |
|  | *Sitophilus oryzae* | XP_030758178.1 |
|  | *Bactrocera dorsalis* | AVD29897.1 |
|  | *Lucilia cuprina* | XP_023295772.1 |
|  | *Drosophila melanogaster* | NP_001285121.1 |
|  | *D. simulans* | KMZ09272.1 |
|  | *D. suzukii* | XP_065724240.1 |
|  | *D. suzukii* | XP_016939330.1 |
| *Mitochondrial GS* | *Contarinia nasturtii* | XP_031618846.1 |
|  | *Sitodiplosis mosellana* | XP_055294997.1 |
|  | *Anopheles stephensi* | XP_035904859.1 |
|  | *B. dorsalis* | AVD29896.1 |
|  | *B. oleae* | XP_014086535.1 |
|  | *A. stephensi* | XP_004530359.1 |
|  | *L. sericata* | XP_037825249.1 |
|  | *Musca domestica* | XP_005181824.1 |
|  | *D. suzukii* | XP_016935075.1 |
|  | *D. melanogaster* | CAA10031.1 |
|  | *D. simulans* | XP_016022876.1 |

**Supplementary figure legends**

Figure S1. Location, distribution of exons and introns, and protein domain and of *RpGS* genes in *Rhopalosiphum padi*. (A) Protein location of the RpGSs in *R. padi*. Genes names and chromosome numbers are showed on the above and below of the bar, respectively. (B) Distribution of exons and introns of *RpGSs* in *R. padi*. The black blocks represent the exons and the black lines represent the introns. (C) The GS protein domain of *R. padi* (Rp), *R. maidis* (Rm) and *Drosophila melanogaster* (Dm) predicted by pfam. Green color rectangles above each of the black lines indicate the GS conserved domains (PLN02284). The scale line at the bottom shows the length of each protein.

Figure S2. Bioinformatics analysis of *RpGSs*. (A) The prediction of hydrophobicity of the RpGSs. (B) The prediction of signal peptides of the RpGSs. (C) The prediction of transmembrane helices of the RpGSs. (D) The distribution of subcellular localizations of RpGSs. Blue indicates predicted subcellular localization.


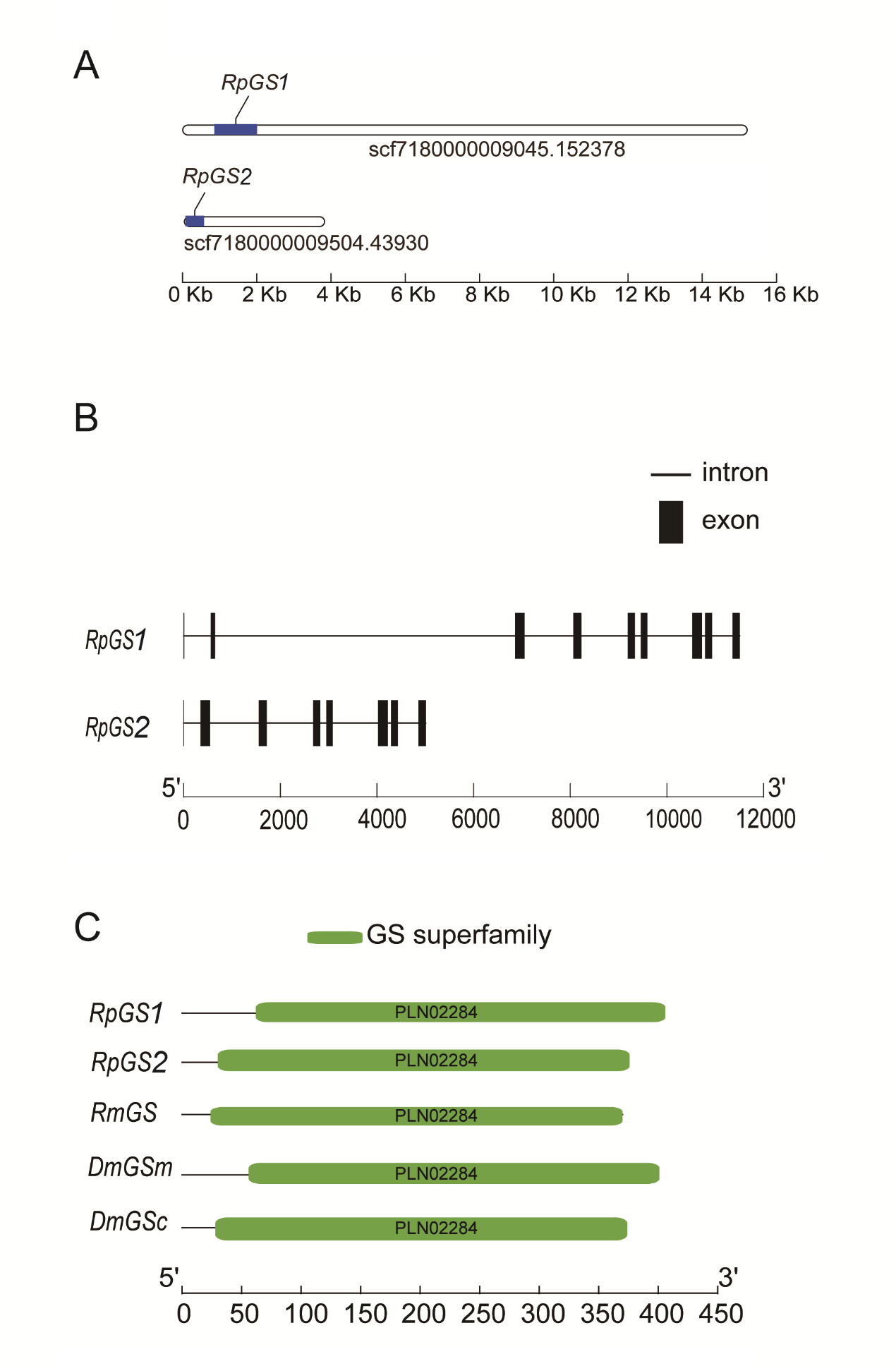


Figure S1


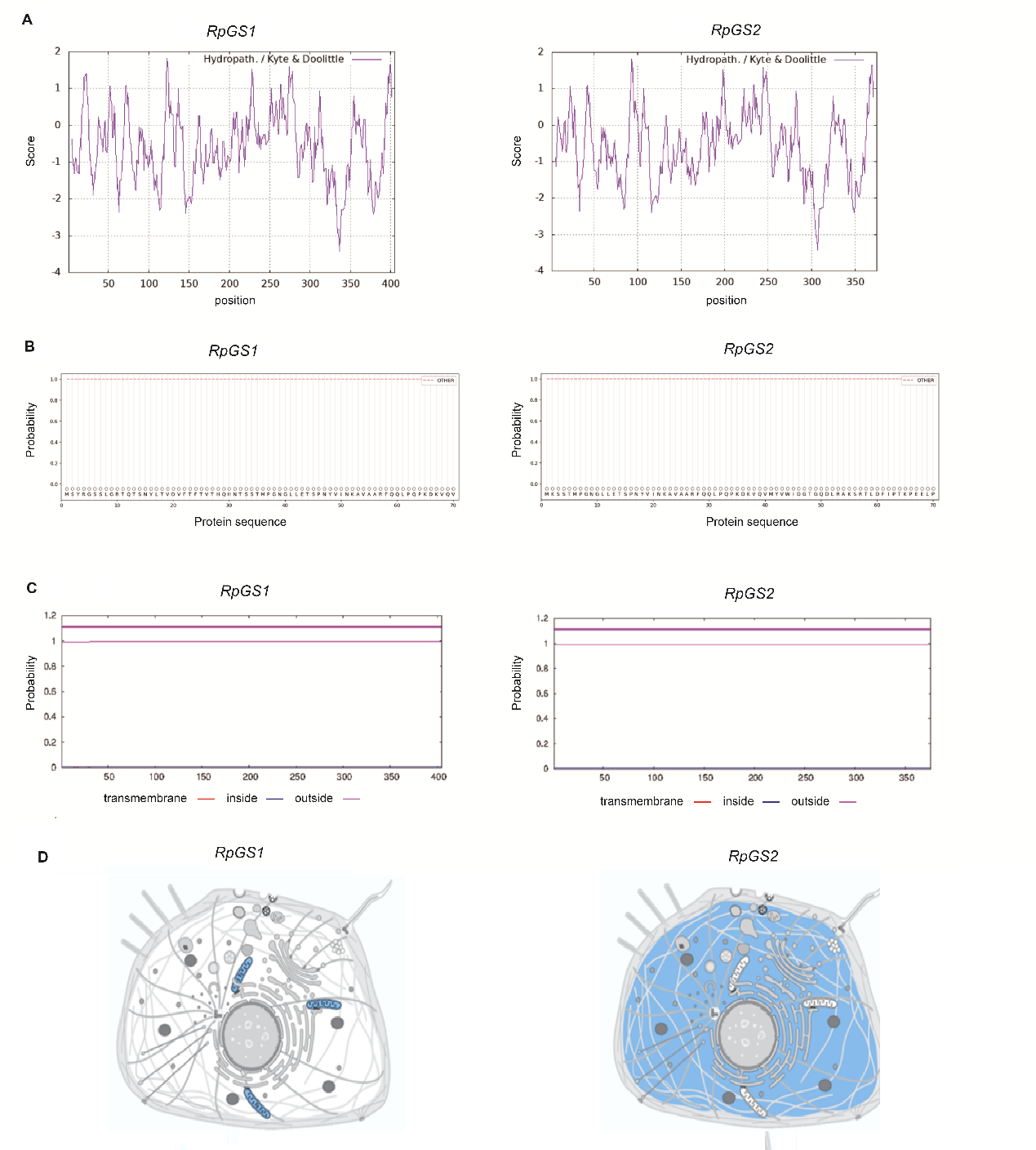


Figure S2
